# Association between triglyceride glucose–body mass index and nonunion in older patients following limb fracture surgery: a multicenter retrospective cohort study

**DOI:** 10.3389/fmed.2026.1747481

**Published:** 2026-04-24

**Authors:** Xinqun Cheng, Zhenbang Yang, Chengsi Li, Puxin Yang, Qingcheng Song, Dongwei Wu, Tianyu Wang, Huilian Sun, Yanbin Zhu, Wei Chen, Yingze Zhang

**Affiliations:** 1 Department of Orthopedic Surgery, The 3rd Hospital of Hebei Medical University, Shijiazhuang, China; 2 Key Laboratory of Biomechanics of Hebei Province, Hebei Orthopedic Research Institute, Shijiazhuang, China

**Keywords:** cohort study, limb fractures, nonunion, older patients, triglyceride glucose–body mass index

## Abstract

**Introduction:**

Metabolic syndrome (MetS) is increasingly recognized as an emerging risk factor affecting fracture healing. We sought to investigate the association between the metabolic abnormality indicator triglyceride glucose–body mass index (TyG–BMI) and postoperative nonunion in older patients with limb fractures.

**Methods:**

In a multicenter retrospective cohort study across five tertiary hospitals, we included older patients (≥60 years) undergoing limb fracture surgery between January 2020 and December 2022. The exposure of interest was the post-injury TyG–BMI, derived from fasting plasma glucose, triglyceride levels, and body mass index. The outcome was nonunion, defined as persistence of the fracture for 9 months or longer without evidence of healing for 3 months. Restricted cubic spline (RCS) model and multivariable logistic regression were used to evaluate the association between TyG–BMI and nonunion. Sensitivity analyses and subgroup analyses were conducted to evaluate the robustness and population heterogeneity of the primary outcome.

**Results:**

Among 8,499 eligible patients [median age 75.0 (67.0, 83.0) years; 60.5% male], 141 cases (1.66%) developed nonunion. RCS revealed a U-shaped, nonlinear relationship between TyG-BMI and nonunion, with risk-protective thresholds at 173.9 and 213.0. Compared with the reference range, both low (<173.9) and high (>213.0) TyG-BMI levels were independently associated with increased risk [TyG-BMI < 173.9: adjusted relative risk (aOR), 1.358; 95% confidence interval (CI), 1.016–1.816; TyG–BMI > 213.0: aOR, 1.233; 95%CI, 1.030–1.475], which remained robust across multiple sensitivity and exploratory analyses. Significant interactions were observed between TyG-BMI levels and perioperative blood transfusion and bone grafting type (P for interaction < 0.05).

**Conclusion:**

Both low and high TyG-BMI levels are significantly associated with increased nonunion risk in older patients following limb fracture surgery, serving as a potential biomarker for risk stratification and individualized management.

## Introduction

Nonunion remains a frequent and debilitating complication in older patients following orthopedic surgery, imposing substantial burdens on both patients and healthcare systems. As reported, patients with nonunion incur 2.6-fold higher direct medical costs and require 2–3 times longer rehabilitation time, with 60–70% reporting persistent pain beyond 6 months and more than half failing to regain pre-injury activity levels within 1 year (1–4). While considerable research has focused on the pathogenesis, treatment modalities, and long-term management strategies for nonunion, efforts to identify reliable and readily accessible early predictive biomarkers remain limited, particularly in the elderly population (5–8).

Existing evidence suggests that metabolic dysfunction is closely associated with altered skeletal remodeling and impaired fracture-healing capacity (9–11). In recent years, metabolic syndrome (MetS) is increasingly recognized as an emerging risk factor affecting fracture healing (12). Older patients are susceptible to the adverse effects of metabolic disorders due to age-related declines in insulin sensitivity and lipid regulation capacity, as well as chronic low-grade inflammation. Insulin resistance, a central pathophysiologic mechanism of MetS, can impair both osteogenic differentiation and physiological angiogenesis (13, 14). Meanwhile, elevated circulating triglyceride-derived fatty acid can induce lipotoxic stress and oxidative damage in bone marrow progenitor cells, while obesity-associated cytokines persistently trigger low-grade inflammatory responses and hinder timely resolution of the inflammatory phase (15–17). Triglyceride glucose -body mass (TyG-BMI), a composite surrogate indicator of metabolic dysfunction, could reflect the cumulative burden of insulin resistance, dyslipidemia, and obesity-related inflammation (18). Therefore, theoretically, a deviant TyG-BMI index could serve as a plausible predictive indicator for nonunion in older patients following limb fracture surgery.

As reported, TyG index and its derivative (e.g., TyG-BMI) are closely associated with the development and adverse outcomes of multiple chronic diseases, such as heart failure, diabetes mellitus, hypertension, and osteoporosis (19–23). Compared with single nutritional or inflammatory markers such as albumin and total lymphocyte count, TyG-BMI may reflect a broader systemic metabolic burden, which is consistent with the growing interest in the potential value of composite lipid and metabolic indices for risk stratification (24). Compared to specialized biomarker assays, costly imaging protocols, and complex clinical scoring systems, TyG-BMI is easily derived from routine laboratory and anthropometric data, offering a simple, cost-effective and easily available alternative. Importantly, it can be calculated at an early stage of admission, making it a potentially valuable tool for early risk stratification in older fracture patients. Given the population aging trend, the substantial clinical and socioeconomic burden of nonunion, and the absence of applicable metabolic predictors, identifying the association between TyG-BMI and nonunion and quantifying its predictive efficacy in older patients becomes both timely and clinically meaningful.

Therefore, this study aimed to identify and quantify the potential association between post-injury TyG-BMI and risk of nonunion in older patients following limb fracture surgery.

## Materials and methods

### Study design and data collection

This retrospective multicenter cohort study was conducted at five tertiary hospitals (two university hospitals and three teaching hospitals) including four Level I and one Level II trauma center, serving a regional population of approximately 28.4 million. Clinical data were manually collected from each institution’s electronic medical records by well-trained researchers independent of clinical care and follow-up was conducted mainly via telephone and outpatient visits. This study complied with the guidelines of Strengthening the Reporting of Observational Studies in Epidemiology (STROBE) and the Declaration of Helsinki (25), and was granted by the local ethical committee [Hebei Medical University Third Hospital: K2024-071-1Cangzhou Hospital of integrated TCM-WM-Hebei: K2024-071-1; Cangzhou People’s Hospital: K2024090-02; Affiliated Hospital of Qingdao University: QYFY WZLL 28864; Baoding No.1 Central Hospital. K(2024) No.183].

### Population

This study screened the patients who underwent surgery for a limb fracture at each participating institutions from January 2020 to December 2022. Eligible patients must be aged 60 years or older, with index limb fracture [diagnosed according to International Classification of Diseases, 9th Revision, Clinical Modification (ICD-9-CM) codes 810.00829.10 18 and confirmed radiographically], underwent surgical treatment, had complete preoperative fasting blood glucose and triglyceride test data along with height and weight records, and completed postoperative follow-up of 12 months or more.

The exclusion criteria included: (1) older fracture (interval between injury and surgery of 21 days or more); (2) stress, pathologic or periprosthetic fractures; (3) fractures of the same limb within 12 months preceding the index fractures; (4) receipt of intravenous glucose solutions, insulin infusion, or systemic glucocorticoid therapy within 12 h prior to blood collection. (5) absence of fasting blood glucose and triglyceride measurements within 48 h post-injury, as well as anthropometric data; (6) acute pancreatitis or systemic infection/sepsis at admission; (7) occurrence of a new fracture during the 12-month follow-up; (8) incomplete follow-up to 12 months.

### Measurement of TyG-BMI

Peripheral venous blood samples are typically collected on the first morning after admission. In cases of emergency surgery, blood is drawn during the initial clinical assessment. Fasting blood glucose (FBG, mmol/L) and triglyceride (TG, mmol/L) levels were measured using an automated biochemical analyzer, following the manufacturer’s protocols. Body weight and height were recorded during hospitalization, and body mass index (BMI, kg/m^2^) was calculated as weight divided by height squared. FBG and TG were measured in mmol/L in the original laboratory system and converted to mg/dL before TyG calculation using standard conversion factors as follows: FBG (mg/dL) = FBG (mmol/L) × 18, and TG (mg/dL) = TG (mmol/L) × 88.57. The TyG index was then calculated as ln [TG (mg/dL) × FBG (mg/dL)/2]. The TyG-BMI was then derived as: TyG-BMI = TyG * BMI. All data were obtained from routine clinical assessments and recorded in the electronic medical record system.

### Outcome

The outcome was defined as nonunion, according to the U. S. Food and Drug Administration (FDA) criteria as a fracture persisting for at least 9 months with no radiographic evidence of healing progression for three consecutive months (26). Nonunion cases were initially identified using ICD-9-CM code 733.82. Each suspected case was subsequently validated through comprehensive medical record review, telephone interviews, and outpatient follow-up assessments. Cases with diagnostic uncertainty were referred to an expert review panel comprising two senior orthopedic surgeons and an experienced radiologist for final adjudication, ensuring diagnostic consistency and accuracy.

### Covariates

We predefined potential confounders that could influence bone healing. Demographic variables included age, sex, place of residence, and surgical history. Lifestyle factors included smoking status and alcohol consumption. Comorbidities comprised hypertension, diabetes, cerebrovascular disease, and heart disease. Laboratory parameters, such as albumin (ALB), red blood cell counts (RBC) and white blood cell counts (WBC), were considered to reflect systemic inflammation and nutritional status. In addition, empirically recognized clinical factors relevant to fracture healing were included, including injury type, surgical site, emergency surgery, wound class, American Society of Anesthesiologists (ASA) class, anesthesia type, operative duration, fixation method, bone grafting and blood transfusion types.

### Statistical analysis

The normality of continuous variables was assessed using the Kolmogorov–Smirnov test. Normally distributed data are presented as mean ± standard deviation and compared with the Student’s *t*-test, whereas non-normally distributed data are presented as median (Q1–Q3) and analyzed using the Mann–Whitney U test. Categorical variables are expressed as numbers (%) and compared using the chi-square or Fisher’s exact test, as appropriate. Missing data were handled by listwise deletion.

A restricted cubic spline (RCS), used four-knots and adjusted by covariables with a univariable *p* < 0.05, was applied to evaluate the nonlinear association between TyG-BMI and the risk of nonunion. A four-knot model was specified, and the cutoff thresholds were determined at the intersection between the spline effect function (RR = 1.00) and the X-axis. Based on these thresholds, patients were categorized into low, middle, and high TyG-BMI groups. Multicollinearity was evaluated through multiple linear regression diagnostics, and variables with a variance inflation factor (VIF) of 3 or more were excluded from further analyses (27). Multivariable binary logistic regression was then used to estimate OR and 95% CI for nonunion in the low and high TyG-BMI groups compared with the middle group, incorporating covariates with a *p* < 0.1 in the univariate analysis for adjustment.

To evaluate the reliability of the indicator and the robustness of the primary outcomes, we performed several sensitivity and exploratory analyses: (1) treating TyG-BMI as a continuous variable; (2) dichotomizing TyG-BMI according to the three-quarter cutoff point; (3) 5% propensity score (PS) trimming of TyG-BMI levels; (4) excluding patients with poor baseline status (ASA ≥ 4); (5) including alkaline phosphatase (ALP) levels as an adjustment covariate; (6) incorporating surgeon experience as a fixed effect; and (7) redefining the dependent variable as a composite outcome of nonunion or delayed union.

Population heterogeneity in the primary outcomes was examined by testing the statistical significance of interaction terms between TyG-BMI levels and the following prespecified subgroups: age (<80 years vs. ≥80 years), gender (male vs. female), fracture type (open vs. closed), blood transfusion type (none vs. allogeneic vs. autologous), and bone grafting type (none vs. autograft vs. allograft).

All statistical analyses were conducted using R software (version 3.6.5; R Foundation for Statistical Computing, Vienna, Austria) and a two-sided *p* <0.05 was considered statistically significant.

## Results

**Figure 1:**
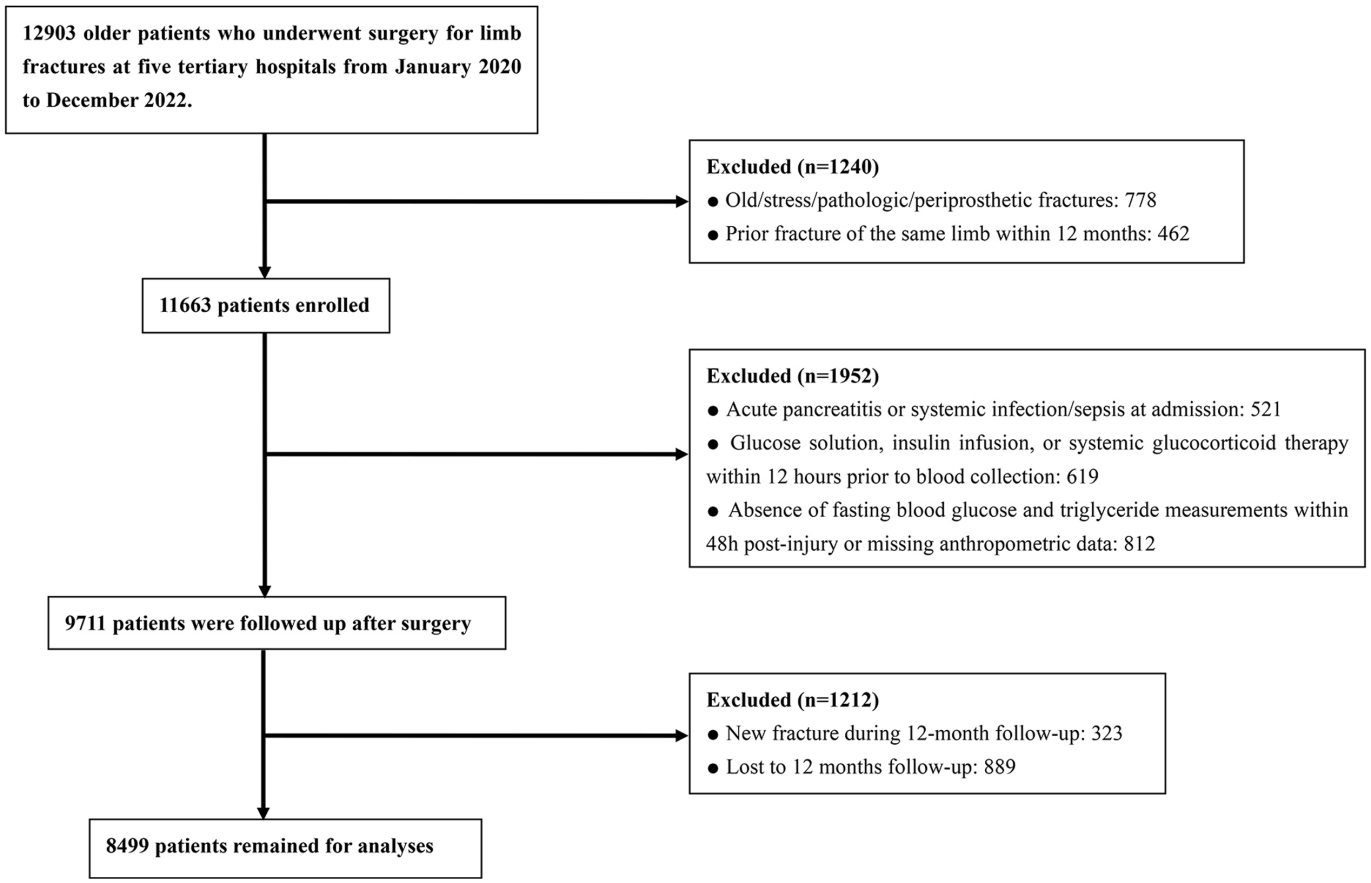
Flowchart for selection of study subjects.

This study initially enrolled 12,903 older patients with limb fractures admitted to the participating hospitals, of whom 4,404 were excluded (Figure 1). Ultimately, 8,499 cases were included in the final analysis; 60.5% were male, and the median age was 75.0 (67.0, 83.0) years. During follow-up, 141 patients developed nonunion, corresponding to a cumulative incidence of 1.66%. At admission, the median fasting blood glucose and triglyceride levels were 5.54 (4.97–6.37) mmol/L and 1.09 (0.79–1.53) mmol/L, respectively, and the median body mass index (BMI) was 25.00 (22.60–27.40) kg/m^2^. Based on these parameters, the median TyG-BMI level was 212.99 (188.61–239.18).

**Figure 2:**
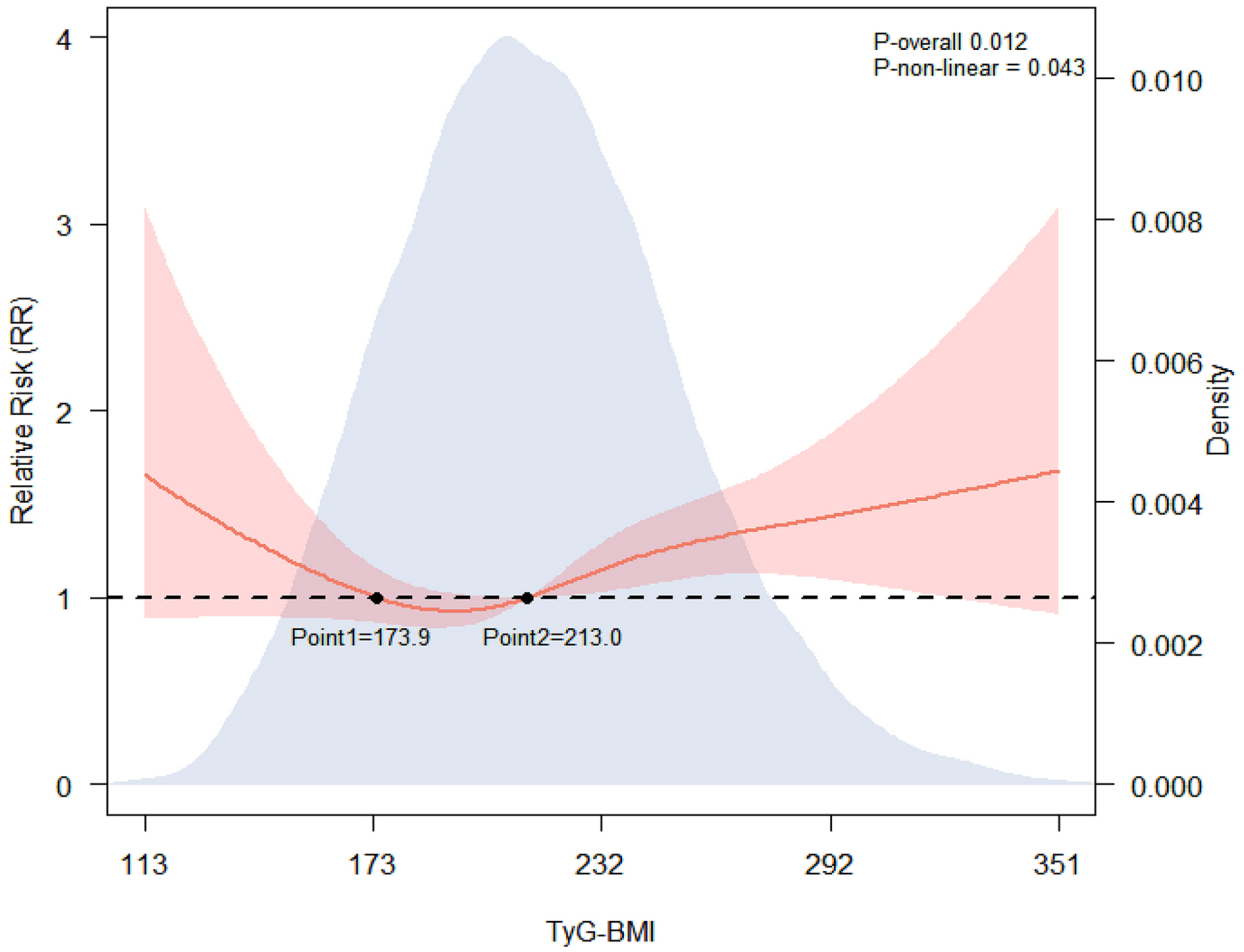
Dose-effect relationship between post-injury TyG-BMI and the risk of nonunion. TyG-BMI, triglyceride-glucose index-body mass index.

RCS analysis revealed a significant U-shaped nonlinear association between post-injury TyG-BMI levels and the risk of nonunion (P for nonlinearity = 0.043; P for overall = 0.012) (Figure 2). which was adjusted by age, gender, place of residence, smoking status, alcohol consumption, hypertension, diabetes mellitus, cerebrovascular disease, heart disease, ALB, RBC, injury type, surgical site, emergency surgery, wound class, ASA class, anesthesia type, operative duration, fixation method, bone grafting type and blood transfusion type (all *p* < 0.05) (Supplementary Table S1). The protective thresholds were identified at 173.9 and 213.0, corresponding to the intersection points of the spline effect function with the baseline (OR = 1.00). Based on these cutoffs, patients were stratified into low (<173.9), middle (173.9–213.0), and high (>213.0) TyG-BMI groups, and significant differences were observed among the three groups at baseline in terms of gender, age, place of residence, alcohol consumption, hypertension, diabetes mellitus, ALB, WBC, RBC, injury type, surgical site, emergency surgery, wound class, ASA class, anesthesia type, operative duration, fixation method, bone grafting type and blood transfusion type (all *p* < 0.05) (Table 1).

**Table 1:** Baseline characteristics of participants.

Characteristic	Low TyG-BMI	Middle TyG-BMI	High TyG-BMI	*p*
Total	788	3,462	4,249	
Nonunion	19 (2.4%)	62 (1.8%)	102 (2.4%)	0.003
Demographic				
Age (≥80 years)	163 (20.7%)	672 (19.4%)	739 (17.4%)	<0.001
Gender (female)	343 (43.5%)	1,385 (40.0%)	1,632 (38.4%)	<0.001
Place of residence				0.005
Country	512 (65.0%)	2,268 (65.5%)	2,702 (63.6%)	
Urban	276 (35.0%)	1,194 (34.5%)	1,547 (36.4%)	
History of surgery	182 (23.1%)	789 (22.8%)	969 (22.8%)	0.002
Lifestyle factors				
Smoking status				0.107
Never smoker	608 (77.2%)	2,610 (75.4%)	3,204 (75.4%)	
Former smoker	153 (19.4%)	699 (20.2%)	858 (20.2%)	
Current smoker	27 (3.4%)	156 (4.5%)	187 (4.4%)	
Alcohol consumption	35 (4.5%)	388 (11.2%)	939 (22.1%)	<0.001
Comorbidities				
Hypertension	95 (12.0%)	595 (17.2%)	1,126 (26.5%)	<0.001
Diabetes mellitus	71 (9.0%)	377 (10.9%)	684 (16.1%)	<0.001
Cerebrovascular disease	66 (8.4%)	312 (9.0%)	387 (9.1%)	0.541
Heart disease	80 (10.1%)	391 (11.3%)	497 (11.7%)	0.061
Laboratory examination				
RBC				<0.001
<Lower limitation	281 (35.7%)	1,177 (34%)	1,139 (26.8%)	
>Higher limitation	7 (0.9%)	42 (1.2%)	76 (1.8%)	
WBC (≥10*10^9^/L)	170 (21.6%)	855 (24.7%)	1,160 (27.3%)	<0.001
ALB (<35 g/L)	209 (26.5%)	869 (25.1%)	918 (21.6%)	<0.001
Treatment				
Injury type				<0.001
Open fracture	161 (20.4%)	647 (18.7%)	676 (15.9%)	
Closed fracture	627 (79.6%)	2,815 (81.3%)	3,573 (84.1%)	
Surgery site				<0.001
Upper limb	288 (36.5%)	1,097 (31.7%)	1,215 (28.6%)	
Lower limb	427 (54.2%)	1935 (55.9%)	2,545 (59.9%)	
Multiple	73 (9.3%)	429 (12.4%)	493 (11.6%)	
Emergency surgery	91 (11.6%)	346 (10.0%)	348 (8.2%)	<0.001
Wound class				<0.001
Clean	668 (84.8%)	2,984 (86.2%)	3,731 (87.8%)	
Clean-contaminated	97 (12.3%)	391 (11.3%)	416 (9.8%)	
Contaminated and dirty	23 (2.9%)	90 (2.6%)	102 (2.4%)	
ASA class				<0.001
I	167 (21.2%)	571 (16.5%)	608 (14.3%)	
II	482 (61.2%)	2,275 (65.7%)	2,834 (66.7%)	
III	133 (16.9%)	578 (16.7%)	769 (18.1%)	
IV&V	6 (0.8%)	38 (1.1%)	42 (1.0%)	
Anesthesia type (general)	551 (69.9%)	2,157 (62.3%)	2,486 (58.5%)	<0.001
Operative duration				<0.001
≤60 min	56 (7.1%)	273 (7.9%)	412 (9.7%)	
61–119 min	348 (44.2%)	1,416 (40.9%)	1,653 (38.9%)	
120–179 min	241 (30.6%)	1,056 (30.5%)	1,270 (29.9%)	
≥180 min	143 (18.1%)	717 (20.7%)	914 (21.5%)	
Blood transfusion type				0.006
None	679 (86.2%)	2,995 (86.5%)	3,680 (86.6%)	
Autologous	14 (1.8%)	97 (2.8%)	98 (2.3%)	
Allogeneic	95 (12.0%)	370 (10.7%)	472 (11.1%)	
Bone grafting type				0.081
None	749 (95%)	3,251 (93.9%)	3,981 (93.7%)	
Autograft	17 (2.2%)	83 (2.4%)	106 (2.5%)	
Allograft	22 (2.8%)	125 (3.6%)	166 (3.9%)	
Fixation method				<0.001
Plate	274 (34.8%)	1,191 (34.4%)	1,398 (32.9%)	
Screw/wire	83 (10.5%)	377 (10.9%)	450 (10.6%)	
Intramedullary nail	118 (15.0%)	419 (12.1%)	472 (11.1%)	
External fixator	313 (39.7%)	1,475 (42.6%)	1929 (45.4%)	

TyG-BMI, triglyceride-glucose index-body mass index; ASA, American society of anesthesiologists; ALB, albumin; WBC, white blood cell; RBC, red blood cell (reference range: Females, 3.5–5.0 *10^^12^/L; males, 4.5–5.5 *10^^12^/L).

Compared with the middle TyG-BMI levels (173.9–213.0), both the low TyG-BMI levels (<173.9) and the high TyG-BMI levels (>213.0) showed significantly increased risks of nonunion (low TyG-BMI levels: OR = 1.344; 95% CI, 1.017–1.775; high TyG-BMI levels: OR = 1.329; 95% CI, 1.120–1.577). After multivariable adjustment, these associations remained statistically significant (low TyG-BMI levels: adjusted OR [aOR] = 1.358; 95% CI, 1.016–1.816; high TyG-BMI levels: aOR = 1.233; 95% CI, 1.030–1.475) (Table 2). No evidence of multicollinearity was found among covariates (Supplementary Table S2).

**Table 2:** Multivariate logistic regression of TyG-BMI and covariates for nonunion.

Characteristic	OR (95%CI)	*P*
TyG-BMI		0.030
Lower group (<173.9)	1.358 (1.016–1.816)	0.038
Higher group (>213.0)	1.233 (1.030–1.475)	0.023
Gender (female)	0.537 (0.427–0.676)	<0.001
Age (≥80 years)	0.656 (0.468–0.922)	0.015
Place of residence (urban)	0.622 (0.507–0.763)	<0.001
Smoking status	–	0.002
Former smoker	0.952 (0.715–1.268)	0.735
Current smoker	1.784 (1.222–2.603)	0.003
Alcohol consumption	1.049 (0.775–1.419)	0.759
Hypertension	0.912 (0.704–1.182)	0.487
Diabetes mellitus	1.102 (0.813–1.495)	0.531
Cerebrovascular disease	0.752 (0.486–1.166)	0.203
Heart disease	0.723 (0.494–1.058)	0.095
WBC (≥10*10^9^/L)	0.851 (0.684–1.060)	0.149
RBC	–	<0.001
<Lower limitation	0.396 (0.305–0.515)	<0.001
>Higher limitation	0.659 (0.317–1.372)	0.265
ALB (<35 g/L)	1.069 (0.805–1.420)	0.644
History of surgery	3.047 (2.547–3.645)	<0.001
Emergency surgery	0.156 (0.089–0.272)	<0.001
Injury type (closed fracture)	0.807 (0.627–1.039)	0.096
Surgery site	–	<0.001
Lower limb	2.157 (1.750–2.658)	<0.001
Multiple	0.133 (0.064–0.277)	<0.001
Anesthesia type (general)	0.724 (0.587–0.893)	0.003
ASA class	–	0.505
II	0.870 (0.687–1.100)	0.244
III	0.945 (0.674–1.324)	0.741
IV&V	0.473 (0.111–2.019)	0.312
Wound class	–	0.007
Clean-contaminated	1.302 (0.979–1.732)	0.070
Contaminated and dirty	2.110 (1.287–3.460)	0.003
Blood transfusion type	–	<0.001
Autologous and allogeneic	3.012 (1.520–5.970)	0.002
Allogeneic	1.487 (1.091–2.027)	0.012
Autologous	3.146 (1.770–5.590)	<0.001
Operative duration	–	0.028
61–119 min	0.737 (0.520–1.045)	0.086
120–179 min	0.722 (0.501–1.041)	0.081
≥180 min	0.976 (0.663–1.437)	0.903
Bone grafting type	–	<0.001
Autograft	25.525 (20.019–32.544)	<0.001
Allograft	6.274 (4.789–8.219)	<0.001
Fixation method	–	<0.001
Screw/wire	0.909 (0.653–1.267)	0.575
Intramedullary nail	3.011 (2.350–3.858)	<0.001
External fixator	0.337 (0.261–0.435)	<0.001

TyG-BMI, triglyceride-glucose index-body mass index; OR, odds ratio; CI, confidence interval; ALB, albumin; RBC, red blood cell; WBC, white blood cell; ASA, American Society of Anesthesiologists classification.

Findings from sensitivity and exploratory analyses generally consistent with the primary findings (Supplementary Table S3), and subgroup analyses revealed significant interactions between TyG-BMI levels and blood transfusion [high TyG-BMI levels: aOR 1.196 (0.983, 1.457) for none, aOR 0.273 (0.043, 1.743) for autologous, aOR 2.252 (1.229, 4.129) for allogeneic, P for interaction = 0.001] as well as bone grafting types [high TyG-BMI levels: aOR 1.149 (0.905, 1.457) for none, aOR 1.296 (0.848, 1.978) for autograft, aOR 1.270 (0.748, 2.157) for allograft, P for interaction < 0.001] (Supplementary Figure S1).

## Discussion

This study is the first to demonstrate a U-shaped, nonlinear association between TyG-BMI and nonunion in older patients following limb fracture surgery. In this multicenter cohort, both lower (<173.9) and higher (>213.0) post-injury TyG-BMI levels were independently associated with an increased risk of postoperative nonunion. This finding suggests that deviation from an intermediate metabolic range may be unfavorable for fracture healing. Clinically, these results support the potential value of TyG-BMI as an accessible early marker for identifying patients at higher risk of impaired healing after limb fracture surgery. The observed association remained generally consistent across sensitivity and exploratory analyses, supporting the robustness of the main findings.

To date, few studies have specifically investigated the association between TyG and its derivative index TyG-BMI and postoperative outcomes in orthopedic patients. In a retrospective cross-sectional study of 3,558 surgically treated patients with osteoporotic fracture, a significant negative nonlinear relationship was observed between the TyG index and bone metabolism markers (*β*-C-terminal telopeptide of type I collagen, procollagen type 1 N-terminal propeptide), suggesting that elevated TyG levels may be detrimental to bone remodeling (28). Similarly, in a cohort of 4,097 osteoarthritis patients, Wang et al. reported a U-shaped, nonlinear association between TyG-BMI and mortality risk, with each 10-unit increase in TyG-BMI level associated with a 3% higher risk of all-cause mortality and a 5% higher risk of cardiovascular mortality, indicating that abnormal TyG-BMI levels may reflect poorer long-term outcomes (29). Consistent with these findings, the present study confirmed that post-trauma TyG-BMI values below 173.9 or above 213.0 were significantly associated with an increased risk of non-union fractures by 35.8 and 23.3%, supporting TyG-BMI as a novel biomarker for predicting fracture healing outcomes in older patients.

The U-shaped relationship between TyG-BMI and the risk of fracture nonunion is biologically plausible. Elevated TyG-BMI levels can reflect the cumulative burden of insulin resistance, dyslipidemia, and obesity-related chronic low-grade inflammation (18). Insulin resistance disrupts PI3K/Akt and mTOR signaling pathways, thereby impairing osteoblast differentiation and angiogenesis (13, 14). Elevated circulating triglyceride-derived fatty acid induce apoptosis in mesenchymal stem cells and trigger lipotoxicity and oxidative stress in osteoprogenitor cells (15, 16), which would be exacerbated by adipose tissue–derived proinflammatory cytokines, such as TNF-*α*, IL-6 (30, 31). Moreover, lipid-induced oxidative stress can damage endothelial cells and hinder microvascular regeneration, further compromising matrix synthesis (32, 33). Conversely, reduced TyG-BMI levels may indicate malnutrition or insufficient energy substrate availability. Nutrient deficiency compromises mitochondrial bioenergetics and protein synthesis in skeletal progenitor cells, while inadequate vitamin D, calcium, or essential amino acids further hinders osteogenesis and delays the transition from inflammation to repair (34–36). In parallel, malnutrition impairs immune competence, reduces leukocyte activity, and prolongs the inflammatory phase, thereby disrupting the tightly regulated coupling of bone resorption and formation (31, 37, 38). These findings suggest that both elevated and reduced TyG-BMI levels create a metabolic microenvironment detrimental to bone formation and remodeling, ultimately increasing the risk of nonunion.

Findings from sensitivity and exploratory analyses are generally consistent with the primary findings, further validating the robustness of our conclusions. At the same time, we also observed a decline in the predictive performance of TyG-BMI in exploratory analyses when delayed union and nonunion were combined as a composite endpoint. In fact, most delayed healing cases ultimately achieve bony union, leading to increased outcome heterogeneity and reduced statistical power, thereby diminishing the predictive specificity of TyG-BMI. Subgroup analysis revealed a stronger association between TyG-BMI and increased nonunion risk in the blood transfusion and bone grafting subgroups, suggesting potential effect modification. This finding indicates that blood transfusion and bone graft recipients may benefit from TyG-BMI-based risk stratification and proactive intervention strategies. However, given the relatively limited sample sizes in the subgroups and the absence of statistical significance threshold adjustment for multiple comparisons, these findings should be interpreted with caution and require further confirmation in subsequent confirmatory studies.

Our findings highlight the potential clinical and public health value of TyG-BMI as a systemic metabolic biomarker in fracture management. In clinical practice, TyG-BMI may help identify patients at higher risk of nonunion, thereby supporting closer metabolic and nutritional assessment after fracture surgery. Clinical evidence indicates that nutritional interventions, such as high-protein energy supplementation and combined vitamin D-calcium regimens, significantly improve bone density and functional recovery in fracture patients (39, 40). From a metabolic perspective, metformin promotes bone-specific microvascular formation (H-type), thereby accelerating fracture healing and improving trabecular remodeling (41). Regarding inflammatory regulation, the combined application of interleukin-1 receptor antagonist (IL-1Ra) with low-dose bone morphogenetic protein-2 (BMP-2) significantly enhances early bone formation and post-repair mechanical strength (42). This series of evidence suggests that nutritional interventions, metabolic regulation, and inflammation-modulating strategies may be relevant to fracture healing. However, given the observational nature of the present study, our findings should not be interpreted as direct evidence supporting these specific interventions. At the public health level, TyG-BMI is a low-cost, readily available, and widely applicable indicator derived from routine laboratory and anthropometric data, which may facilitate early risk screening in fracture populations.

There are several limitations should be noted. First, the retrospective design inherently carries risks of selection bias, information bias, and unmeasured confounding. Although VIF analysis indicated that multicollinearity was unlikely to introduce bias in effect estimates, it cannot exclude residual confounding or model misspecification. Thus, prospective or randomized controlled studies remain warranted to validate these findings in the future. Second, although suspected nonunion cases were further validated through medical record review, telephone follow-up, and outpatient assessment, and diagnostically uncertain cases were adjudicated by the same fixed expert panel, formal inter-rater reliability statistics such as kappa were not available because this retrospective process was based on consensus review rather than prospectively archived independent duplicate ratings. Therefore, detection bias of the outcome cannot be completely eliminated. Third, TyG-BMI was calculated using fasting blood glucose and triglyceride levels measured during the early post-injury period, typically on the first morning after admission or during the initial assessment in emergency cases. Therefore, post-injury TyG-BMI in this study may reflect not only the patients’ underlying chronic metabolic status but also the acute metabolic stress response induced by trauma. To minimize this bias, we excluded patients who had received intravenous glucose infusion, insulin infusion, or systemic glucocorticoid therapy within 12 h before blood collection, and included only those who underwent fasting glucose and triglyceride testing within 48 h after injury. In addition, we performed a sensitivity analysis excluding patients with an injury-to-surgery time of less than 24 h, and the main association remained materially unchanged. Nevertheless, trauma-related stress responses, peri-injury fasting status, and unrecorded pre-transfer interventions may still have influenced these measurements. Finally, although the multicenter design minimized inter-institutional variability, all participating hospitals were tertiary referral centers (including four Level I trauma centers). Consequently, study subjects may have presented with more complex fracture types and received more advanced perioperative treatments, limiting the generalizability of findings to other healthcare settings. Therefore, future studies should incorporate data from healthcare facilities of varying levels and more diverse patient populations to enhance external validity and clinical applicability.

## Conclusion

This study demonstrates that both low and high post-injury TyG-BMI levels are significantly associated with an increased risk of nonunion in older patients following limb fracture surgery. It may serve as a novel biomarker for guiding nonunion risk stratification and individualized management.

## Data Availability

The raw data supporting the conclusions of this article will be made available by the authors, without undue reservation.
