# Supplementary material for: Association between triglyceride glucose–body mass index and nonunion in older patients following limb fracture surgery: a multicenter retrospective cohort study
Source: Front Med (Lausanne). 2026 Apr 24;13:1747481. doi: 10.3389/fmed.2026.1747481 (PMC13152767; doi:10.3389/fmed.2026.1747481)
Supplement: Supplementary file 1 [file Data_sheet_1.docx]

**Table S1.** Baseline characteristics of the patients.

| **Characteristic** | **Patients without nonunion** | **Patients with nonunion** | **p** |
| --- | --- | --- | --- |
| **Total** | 8358 | 141 |  |
| **Demographic** |  |  |  |
| Age (≥80 years) | 1396(16.7%) | 12(8.5%) | <0.001 |
| Gender (female) | 3235(38.7%) | 32(22.7%) | <0.001 |
| BMI (kg/m^2^) |  |  | 0.007 |
| <18.5 | 150(1.8%) | 4(2.8%) |  |
| 18.5-23.9 | 3085(36.9%) | 43(30.5%) |  |
| 24-27.9 | 3477(41.6%) | 60(42.6%) |  |
| ≥28 | 1646(19.7%) | 34(24.1%) |  |
| Place of residence |  |  | <0.001 |
| Country | 5366(64.2%) | 108(76.6%) |  |
| Urban | 2992(35.8%) | 33(23.4%) |  |
| History of surgery | 1931(23.1%) | 75(53.2%) | <0.001 |
| **Lifestyle factors** |  |  |  |
| Smoking status |  |  | <0.001 |
| Never smoker | 6335(75.8%) | 93(66%) |  |
| Former smoker | 1672(20%) | 36(25.5%) |  |
| Current smoker | 351(4.2%) | 12(8.5%) |  |
| Alcohol consumption | 1321(15.8%) | 32(22.7%) | <0.001 |
| **Comorbidities** |  |  |  |
| Hypertension | 1270(15.2%) | 30(21.3%) | <0.001 |
| Diabetes mellitus | 736(8.8%) | 19(13.5%) | 0.001 |
| Cerebrovascular disease | 326(3.9%) | 14(9.9%) | <0.001 |
| Heart disease | 443(5.3%) | 16(11.3%) | <0.001 |
| **Laboratory examination** |  |  |  |
| RBC |  |  | <0.001 |
| ＜lower limitation | 1730(20.7%) | 42(29.8%) |  |
| ＞higher limitation | 117(1.4%) | 2(1.4%) |  |
| WBC (≥10*10^9^/L) | 1906(22.8%) | 36(25.5%) | 0.091 |
| ALB (<35 g/L) | 6386(76.4%) | 114(80.9%) | 0.008 |
| TyG | 8.5 [8.2-8.9] | 8.6 [8.2-9.0] | <0.001 |
| TyG-BMI | 212.9 [188.6-238.9] | 218.6 [193.1-247.0] | <0.001 |
| **Treatment** |  |  |  |
| Injury type |  |  | <0.001 |
| Open fracture | 1446(17.3%) | 35(24.8%) |  |
| Closed fracture | 6912(82.7%) | 106(75.2%) |  |
| Surgery site |  |  | <0.001 |
| Upper limb | 2566(30.7%) | 39(27.7%) |  |
| Lower limb | 4797(57.4%) | 100(70.9%) |  |
| Multiple | 995(11.9%) | 2(1.4%) |  |
| Emergency surgery | 777(9.3%) | 3(2.1%) | <0.001 |
| Wound class |  |  | <0.001 |
| Clean | 7279(87.1%) | 114(80.9%) |  |
| Clean-contaminated | 878(10.5%) | 22(15.6%) |  |
| Contaminated & Dirty | 201(2.4%) | 5(3.5%) |  |
| ASA class |  |  | <0.001 |
| I | 1463(17.5%) | 19(13.5%) |  |
| II | 5491(65.7%) | 92(65.2%) |  |
| III | 1312(15.7%) | 27(19.1%) |  |
| IV&V | 92(1.1%) | 3(2.1%) |  |
| Anesthesia type (General) | 5090(60.9%) | 100(70.9%) | <0.001 |
| Operative duration |  |  | <0.001 |
| ≤60 min | 736(8.8%) | 10(7.1%) |  |
| 61-119 min | 3376(40.4%) | 45(31.9%) |  |
| 120-179 min | 2524(30.2%) | 42(29.8%) |  |
| ≥180 min | 1722(20.6%) | 44(31.2%) |  |
| Blood transfusion type |  |  | <0.001 |
| None | 7238(86.6%) | 111(78.7%) |  |
| Autologous | 201(2.4%) | 7(5%) |  |
| Allogeneic | 919(11%) | 23(16.3%) |  |
| Bone grafting Type |  |  | <0.001 |
| None | 7915(94.7%) | 78(55.3%) |  |
| Autograft | 159(1.9%) | 42(29.8%) |  |
| Allograft | 284(3.4%) | 21(14.9%) |  |
| Fixation method |  |  | <0.001 |
| Plate | 2867(34.3%) | 57(40.4%) |  |
| Screw/Wire | 1154(13.8%) | 9(6.4%) |  |
| Intramedullary nail | 3042(36.4%) | 63(44.7%) |  |
| External fixator | 1295(15.5%) | 12(8.5%) |  |

Abbreviations: TyG, triglyceride-glucose; BMI, body mass index; TyG-BMI, triglyceride glucose-body mass index; ASA, American society of anesthesiologists; ALB, albumin; WBC, white blood cell; RBC, red blood cell (reference range: Females, 3.5-5.0 *10^^12^/L; males, 4.5-5.5 *10^^12^/L).

**Table S2.** Diagnostic test for collinearity.

| **Characteristic** | **Standardized Coefficient** | **t** | **P** | **Collinearity Statistics** | |
| --- | --- | --- | --- | --- | --- |
|  | **Beta** |  |  | **Tolerance** | **VIF** |
| (Constant) |  | 8.333 | ＜0.001 |  |  |
| TyG-BMI | 0.019 | 3.193 | 0.001 | 0.940 | 1.064 |
| Age | -0.014 | -2.008 | 0.045 | 0.699 | 1.431 |
| Gender | -0.037 | -5.639 | ＜0.001 | 0.733 | 1.364 |
| Place of residence | -0.030 | -5.238 | ＜0.001 | 0.966 | 1.036 |
| Smoking status | 0.021 | 2.761 | 0.006 | 0.552 | 1.811 |
| Alcohol consumption | -0.010 | -1.402 | 0.161 | 0.578 | 1.731 |
| Hypertension | -0.005 | -0.804 | 0.421 | 0.820 | 1.220 |
| Diabetes mellitus | -0.008 | -1.342 | 0.180 | 0.918 | 1.089 |
| Cerebrovascular disease | -0.009 | -1.460 | 0.144 | 0.871 | 1.148 |
| Heart disease | -0.013 | -2.081 | 0.037 | 0.868 | 1.153 |
| WBC | -0.019 | -3.179 | 0.001 | 0.910 | 1.099 |
| ALB | -0.003 | -0.507 | 0.612 | 0.677 | 1.477 |
| RBC | -0.046 | -7.078 | ＜0.001 | 0.760 | 1.315 |
| History of surgery | 0.101 | 17.748 | ＜0.001 | 0.967 | 1.034 |
| Emergency surgery | -0.059 | -8.350 | ＜0.001 | 0.632 | 1.582 |
| Injury type | -0.037 | -4.947 | ＜0.001 | 0.550 | 1.817 |
| Surgery site | -0.002 | -0.389 | 0.697 | 0.810 | 1.235 |
| Anesthesia type | -0.034 | -5.466 | ＜0.001 | 0.811 | 1.233 |
| ASA class | -0.009 | -1.433 | 0.152 | 0.829 | 1.207 |
| Wound class | 0.032 | 4.473 | ＜0.001 | 0.636 | 1.572 |
| Operative duration | 0.012 | 1.913 | 0.056 | 0.765 | 1.307 |
| Fixation method | -0.040 | -6.637 | ＜0.001 | 0.871 | 1.149 |
| Bone grafting Type | 0.168 | 29.470 | ＜0.001 | 0.975 | 1.026 |
| Blood transfusion type | 0.026 | 4.086 | ＜0.001 | 0.773 | 1.294 |

Abbreviations: TyG-BMI, triglyceride-glucose index-body mass index; VIF, variance inflation factor; ALB, albumin; RBC, red blood cell; WBC, white blood cell; ASA, American Society of Anesthesiologists classification.

**Table S3.** Sensitivity and exploratory analyses

| **No.** | **TyG-BMI level** | **Unadjusted Model** | | **Adjusted Model** | |
| --- | --- | --- | --- | --- | --- |
|  |  | **OR 95%CI** | **P** | **OR 95%CI** | **P** |
| #1 | - | 1.004 (1.002 - 1.006) | < 0.001 | 1.003 (1.000 - 1.1005) | 0.025 |
| #2 | >Q3 | 1.400 (1.182 – 1.658) | < 0.001 | 1.252 (1.046 - 1.499) | 0.014 |
| #3 | 173.9-213.0 | - | 0.004 | - | 0.039 |
|  | <173.9 | 1.325 (0.993 - 1.769) | 0.056 | 1.359 (1.006 - 1.836) | 0.046 |
|  | >213.0 | 1.332 (1.118 - 1.587) | 0.001 | 1.227 (1.021 - 1.476) | 0.029 |
| #4 | 173.9-213.0 | - | 0.003 | - | 0.028 |
|  | <173.9 | 1.346 (1.018 - 1.778) | 0.037 | 1.366 (1.022 - 1.827) | 0.035 |
|  | >213.0 | 1.331 (1.121 - 1.579) | 0.001 | 1.235 (1.031 - 1.479) | 0.022 |
| #5 | 173.9-213.0 | - | 0.003 | - | 0.027 |
|  | <173.9 | 1.344 (1.017-1.775) | 0.038 | 1.388 (1.000-1.789) | 0.050 |
|  | >213.0 | 1.329 (1.120-1.577) | 0.001 | 1.249 (1.043-1.496) | 0.015 |
| #6 | 173.9-213.0 | - | 0.003 | - | 0.008 |
|  | <173.9 | 1.344 (1.017-1.775) | 0.038 | 1.413 (1.052-1.897) | 0.021 |
|  | >213.0 | 1.329 (1.120-1.577) | 0.001 | 1.299 (1.081-1.560) | 0.022 |
| #7 | 173.9-213.0 | - | 0.776 | - | 0.659 |
|  | <173.9 | 1.047 (0.881-1.244) | 0.603 | 1.027 (0.857-1.231) | 0.770 |
|  | >213.0 | 1.033 (0.934-1.143) | 0.528 | 1.051 (0.944-1.170) | 0.361 |
| #8 | 173.9-213.0 | - | 0.019 | - | 0.026 |
|  | <173.9 | 1.348 (1.001-1.815) | 0.049 | 1.372 (1.018-1.849) | 0.037 |
|  | >213.0 | 1.231 (1.024-1.480) | 0.007 | 1.244 (1.035-1.495) | 0.020 |

Abbreviations: TyG-BMI, triglyceride-glucose index-body mass index; OR, odds ratio; CI=confidence interval.

# 1 Treating TyG–BMI as a continuous variable.

# 2 Dichotomizing TyG–BMI at the third quartile (Q3).

# 3 5% propensity score (PS) trimming of TyG-BMI.

# 4 Excluding patients with ASA class of 4 or more.

# 5 Including alkaline phosphatase (ALP) as a new covariate for analysis.

# 6 Incorporating surgeon experience as a fixed effect.

# 7 Redefining the dependent variable as a composite outcome of nonunion or delayed union.

# 8 Excluding patients with an injury-to-surgery time of less than 24 hours.

**Figure S1**. The association of TyG-BMI with the risk of nonunion in various subgroups.


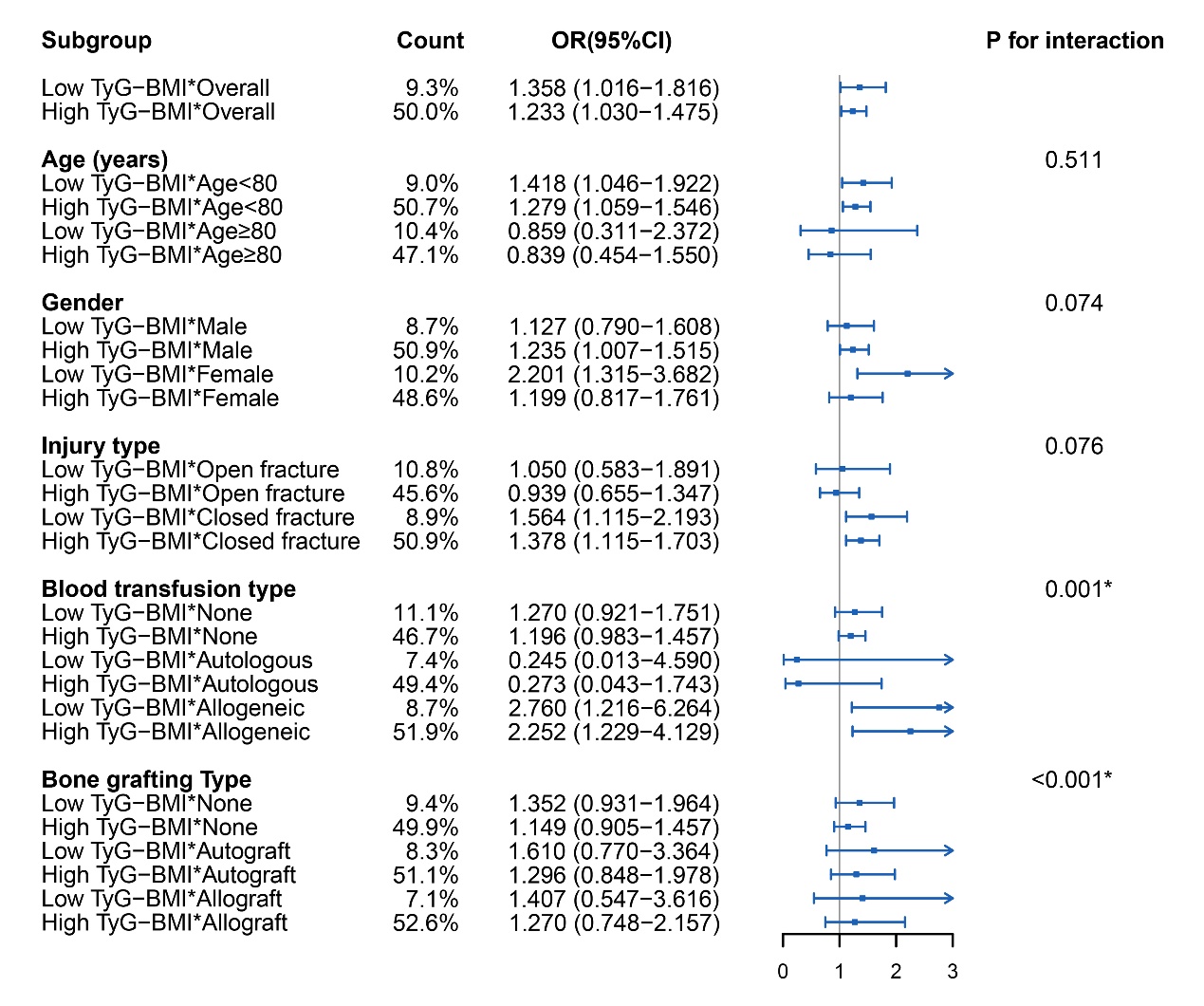
Abbreviations: TyG-BMI, triglyceride-glucose index-body mass index; OR, odds ratio; CI, confidence interval.
